# Biological Safety and Functional Properties of Açaí Seed Extract: Antioxidant Capacity and Prebiotic Potential

**DOI:** 10.1111/1750-3841.71293

**Published:** 2026-07-14

**Authors:** Marcelo Pereira da Silva Junior, Amanda de Jesus Alves Miranda, Ana Laura Gagliardi, Isabelle Guerreiro Moreira, Maria Eduarda Santos Camaceti, Mateus Kawata Salgaço, Kátia Sivieri, Cláudia Quintino da Rocha, Flávia Aparecida Resende

**Affiliations:** ^1^ Department of Biological Sciences and Health University of Araraquara (UNIARA) Araraquara São Paulo Brazil; ^2^ Department of Chemistry Federal University of Maranhão (UFMA) São Luís Maranhão Brazil

**Keywords:** açaí, biological properties, *Euterpe oleracea*, plant extracts, toxicological evaluation

## Abstract

Açaí (*Euterpe oleracea*) is widely recognized as a functional food due to its nutritional value and abundance of bioactive compounds. Its seeds, representing approximately 80%–95% of the fruit mass, constitute an abundant agro‐industrial byproduct with promising biotechnological potential. This study aimed to chemically characterize a 70% ethanolic extract obtained from *E. oleracea* seeds through liquid chromatography–mass spectrometry (LC–MS) annotation and total phenolic content (TPC) determination, as well as to investigate its antioxidant activity, prebiotic potential, cytotoxicity, and mutagenic safety profile. The extract was obtained by percolation using 70% ethanol. Antioxidant activity was evaluated by the DPPH assay, while prebiotic potential was assessed through submerged in vitro fermentation with *Lactiplantibacillus plantarum* LP90, monitoring bacterial growth and medium acidification. Cytotoxicity was evaluated in Caco‐2 cells using a resazurin assay, and mutagenicity was investigated using the micronucleus and Ames tests. LC–MS annotation revealed a phenolic‐rich profile, mainly composed of procyanidins. The extract showed a TPC of 123.12 ± 5.70 mg GAE/g dry weight and significant antioxidant activity (EC_50_ = 11.79 ± 0.9 µg/mL). Additionally, the extract promoted *L. plantarum* growth and reduced medium pH, suggesting fermentable substrate potential. The extract also demonstrated low cytotoxicity (IC_50_ = 704.4 ± 41.8 µg/mL) and no mutagenic effects. These findings support the valorization of *E. oleracea* seeds as a potential source of bioactive compounds for functional and nutraceutical applications.

## 1 Introduction

Medicinal plants and their derived extracts have been used for centuries in traditional medicine and continue to represent an important source of bioactive compounds for modern therapeutics. Their broad availability, structural diversity, and relatively low incidence of adverse effects compared with synthetic drugs have stimulated extensive scientific investigation (Uma et al. 2017).

Within this context, the Arecaceae family stands out due to its wide geographical distribution and remarkable biochemical diversity. This family comprises approximately 2600 species distributed among 181 genera worldwide, with several species occurring in Brazilian transitional biomes between the Amazon, Cerrado, and Caatinga (de Souza et al. 2020). Genera such as *Geonoma*, *Syagrus*, *Bactris*, *Attalea*, *Allagoptera*, *Astrocaryum*, and *Euterpe* are among the most representative groups (Morais et al. 2022). Many of these palms are valued not only for their nutritional importance but also for their rich composition of bioactive compounds, including carotenoids, polyunsaturated fatty acids, tocopherols, phenolic compounds, fibers, and minerals. Such biochemical diversity has enabled their use in food production, folk medicine, bioenergy generation, fiber production, and traditional handicrafts (de Souza et al. 2020).

Among these species, *Euterpe oleracea* Mart., popularly known as açaí, has received considerable scientific and commercial attention due to the high nutritional and functional value of its fruits. This species is widely distributed across the Amazon basin, particularly in Brazil, Venezuela, and Guyana, with large‐scale cultivation occurring in the estuarine regions of the Amazon River (Da Silva et al. 2023; Laurindo et al. 2023). Beyond its growing international market as a functional food, açaí plays a central role in the diet of Amazonian populations, especially in the Brazilian state of Pará, where it is consumed daily as a staple food accompanying fish, shrimp, or cassava‐based products. In this region, açaí represents an important source of energy and nutrients and contributes significantly to the livelihoods of local communities involved in its production and commercialization (Silveira et al. 2023).

The health‐promoting properties of açaí have been mainly attributed to its high concentration of phenolic compounds, particularly anthocyanins, proanthocyanidins, and other flavonoids, which are distributed across different parts of the plant, including the leaves, pulp, peel, and seeds (Laurindo et al. 2023; Silveira et al. 2023). However, despite the extensive consumption of the fruit pulp, the seeds represent the largest fraction of the fruit, accounting for approximately 80%–95% of its total mass. During industrial processing, these seeds are typically discarded, generating large quantities of agro‐industrial residues in producing regions (Melo et al. 2021; Laurindo et al. 2023). The valorization of this biomass has therefore emerged as an important strategy within the framework of sustainable bioprocessing and circular economy, aiming to reduce environmental impacts while creating new value‐added products.

Extracts obtained from this byproduct have demonstrated significant antioxidant capacity in different experimental models (Rodrigues et al. 2006; Barros et al. 2015; Martinez et al. 2018; G. R. Martins et al. 2020, 2022; Previtalli‐Silva et al. 2024). In addition, anti‐inflammatory (Xavier et al. 2021; da Silva Monteiro et al. 2021, 2024; Genovese et al. 2022), antihypertensive (da Silva et al. 2020; de Andrade Soares et al. 2023; de Oliveira et al. 2025), renoprotective (da Costa et al. 2017; da Silva Cristino Cordeiro et al. 2018; Monteiro et al. 2022), and antidiabetic effects (de Bem, Costa, et al. 2018; de Bem, da Costa, et al. 2018) have been reported in experimental models. These biological effects have been associated with improvements in oxidative stress, inflammatory responses, lipid metabolism, renal function, and insulin signaling pathways (de Bem, Costa, et al. 2018; de Bem, da Costa, et al. 2018; Santos et al. 2020). Furthermore, cytotoxic effects of açaí seed extracts have been described in several cancer cell lines, including breast, lung, and prostate cancer models, involving mechanisms related to reactive oxygen species generation, autophagy activation, apoptosis induction, and cell‐cycle arrest (D. S. Freitas et al. 2017; Martinez et al. 2018; Silva et al. 2021; Muniz Filho et al. 2023).

Beyond their pharmacological properties, açaí seeds have also attracted attention for diverse technological applications. Previous studies have explored their use in the production of activated carbon for dye adsorption (Pessôa et al. 2019), biochar for soil improvement (Sato et al. 2020), fermentation substrates for *Saccharomyces cerevisiae*, and nanoporous carbon materials for CO_2_ capture (de Souza et al. 2020). These approaches reinforce the importance of transforming a widely available agro‐industrial residue into valuable bioproducts, contributing to sustainable resource management (L. S. Martins et al. 2021).

Despite the growing number of studies describing the potential applications of *E. oleracea* derivatives, important gaps remain regarding the safety characterization of these products, particularly considering the chemical variability commonly observed in natural extracts and its potential impact on biological activity.

Therefore, the present study aimed to characterize the chemical profile of an açaí seed extract by liquid chromatography–mass spectrometry (LC–MS), quantify its total phenolic content (TPC), and investigate the relationship between its phenolic composition and its antioxidant and prebiotic activities. The study focused on a 70% ethanolic extract obtained from *E. oleracea* seeds, characterized by a high proanthocyanidin content. In addition, its cytotoxicity was assessed in intestinal Caco‐2 cells, together with its mutagenic potential at both the chromosomal and gene mutation levels. Overall, the findings provide scientific evidence supporting the safe valorization and potential application of this agro‐industrial byproduct in nutraceutical, biotechnological, and functional food fields.

## 2 Materials and Methods

### 2.1 Preparation and Chemical Characterization of *E. oleracea* Seed Extract

Açaí seeds were collected in Axixá, Maranhão State, Brazil, within the Amazon region (2°50′19.4″S, 44°03′29.6″W). The seeds were dried in a forced‐air oven at 40°C–50°C and ground using a knife mill. For the extraction, 100 g of dried açaí seeds were subjected to exhaustive percolation with 2 L of 70% ethanol (v/v). The extraction was carried out in 24‐h cycles, with 150 mL of fresh 70% ethanol added at each cycle. After extraction, the liquid extract was filtered and concentrated under reduced pressure using a rotary evaporator at temperatures below 40°C. The concentrated extract was subsequently freeze‐dried for 48 h at −95°C under a pressure of 12 µHg.

The extract was analyzed using a Shimadzu Prominence liquid chromatography system with two Shimadzu LC‐20AD automatic injector (SIL‐20A HT) pumps. A C18 Kinetex (250 × 4.6 mm, 5 µm) column was used in the analyses. The mobile phase was acidified with ultrapure water (0.1% HCOOH). HPLC‐grade methanol, also acidified (0.1% HCOOH), was used at a flow rate of 1.0 mL/min, with the methanol gradient increasing as follows: 5%–10% methanol in 5–30 min; 10%–30% methanol in 30–40 min; 30%–60% methanol in 40–50 min; and 60%–80% methanol in 50–60 min. The injection volume was 20.0 µL. The LC was coupled to a mass spectrometer (Amazon X, Bruker, Billerica, MA, USA) equipped with electrospray ionization (ESI) and an ion‐trap (IT) type analyzer in negative mode, under the following conditions: capillary voltage of 5 kV, capillary temperature of 325°C, entrainment gas (N2) flow of 12 L/min, and nitrogen nebulizer pressure of 10 psi. The acquisition range was *m*/*z* 50–1500, with two or more events.

### 2.2 Determination of TPC of Açaí Seed Extract by the Folin–Ciocalteu Colorimetric Method

The TPC of the açaí seed extract was determined and expressed as milligrams of gallic acid equivalents per gram of dry weight (mg GAE/g DW), according to the Folin–Ciocalteu colorimetric method described by Waterhouse (2001). Briefly, a 20‐µL aliquot of the extract or gallic acid standard solution (50–500 mg/L) was added to 1.58 mL of distilled water, followed by the addition of 100 µL of the Folin–Ciocalteu reagent. The mixture was vortexed and allowed to stand at room temperature for 8 min. Subsequently, 300 µL of a 20% (w/v) aqueous sodium carbonate solution was added, and the samples were vortexed again and incubated at room temperature for 2 h. The absorbance was then measured at 765 nm using a Shimadzu UV–Vis spectrophotometer (UV‐160U, Kyoto, Japan) with 1 cm disposable cuvettes. All analyses were performed in triplicate.

### 2.3 Antioxidant Activity (DPPH Assay)

The antioxidant activity of the *E. oleracea* seed extract was determined using the 2,2‐diphenyl‐1‐picrylhydrazyl (DPPH•) radical scavenging assay in 96‐well microplates, according to Rufino et al. (2010), with modifications.

Briefly, 20 µL of extract solution (1.5–50 µg/mL), previously solubilized in ethanol containing 0.1% DMSO, was mixed with 200 µL of DPPH solution (0.05 mg/mL in ethanol). The negative control consisted of ethanol and DPPH solution, while blanks containing extract and ethanol were used to correct for background absorbance. All experiments were performed in quadruplicate.

After incubation for 20 min at room temperature in the dark, absorbance was measured at 517 nm using a UV–Vis spectrophotometer. Radical scavenging activity was expressed as a percentage of inhibition relative to the control. The effective concentration required to reduce 50% of the DPPH radical (EC_50_) was calculated by linear regression analysis of the inhibition curve using GraphPad Prism 7.0 (GraphPad Software, San Diego, CA, USA).

Antioxidant capacity was also expressed as Trolox equivalent antioxidant capacity (TEAC). A calibration curve was constructed using Trolox ((±)‐6‐hydroxy‐2,5,7,8‐tetramethylchromane‐2‐carboxylic acid; 100–600 µmol/L), and results were expressed as micromoles of Trolox equivalents per gram of sample.

### 2.4 Prebiotic Activity

The prebiotic potential of the *E. oleracea* seed extract was evaluated based on its ability to stimulate the growth of *Lactiplantibacillus plantarum* LP90, according to Mashudin et al. (2024).

The bacterial strain was reactivated in Man Rogosa and Sharpe (MRS) broth (2% v/v) and incubated at 37°C under agitation (120 rpm) for 48 h. Bacterial culture was then centrifuged (2000 rpm, 20 min, 4°C), resuspended in sterile ultrapure water, and standardized to an optical density of 0.8 at 600 nm (OD_600_).

To evaluate the ability of the extract to support probiotic bacterial growth, a carbohydrate‐free MRS medium was prepared by replacing glucose with *E. oleracea* seed extract (20 g/L), while maintaining the remaining components unchanged. The following conditions were evaluated: (i) standard MRS containing 2% glucose, (ii) glucose‐free MRS (modified MRS), (iii) glucose‐free MRS supplemented with 20 g/L of açaí seed extract previously sterilized by filtration (0.22 µm membrane, Millipore), and (iv) glucose‐free MRS supplemented with 2 g/L inulin (positive control).

Each flask containing 50 mL of the respective medium was inoculated with 0.5 mL of the standardized bacterial suspension. Fermentations were carried out at 37°C and 120 rpm for 24 h. Aliquots were collected at 0, 2, 4, 6, 8, and 24 h.

Bacterial growth was monitored by measuring optical density at 600 nm using a UV–Vis spectrophotometer, and pH values were recorded at the same time points to assess medium acidification during fermentation.

### 2.5 Cytotoxic Activity

Cytotoxicity was evaluated in human colorectal adenocarcinoma cells (Caco‐2, BCRJ No. 0059) using the resazurin reduction assay, according to Page et al. (1993).

Cells were cultured in Dulbecco's Modified Eagle Medium (DMEM; Gibco, Waltham, MA, USA) supplemented with 20% fetal bovine serum (FBS) at 37°C in a humidified atmosphere containing 5% CO_2_.

For the assay, cells were seeded in 96‐well plates at a density of 1 × 10^4^ cells/well and incubated for 24 h to allow cell attachment. The extract, previously solubilized in DMEM containing 0.1% DMSO (v/v), was tested at final concentrations ranging from 31.25 to 1000 µg/mL. Negative control (culture medium), solvent control (0.1% DMSO), and positive control (50% DMSO) were included. All treatments were performed in triplicate in three independent experiments.

After 24 h of exposure, the medium was removed, and 50 µL of resazurin solution (0.01% w/v) was added to each well, followed by incubation for 4 h. Fluorescence was measured using a Synergy H1 microplate reader (BioTek) at excitation and emission wavelengths of 560 and 590 nm, respectively. Cell viability was expressed as a percentage relative to untreated control cells. The half‐maximal inhibitory concentration (IC_50_) was determined by nonlinear regression using GraphPad Prism 7.0 (GraphPad Software, San Diego, CA, USA).

### 2.6 Mutagenic Activity

#### 2.6.1 Micronucleus Assay

The micronucleus assay was performed in Caco‐2 cells to evaluate chromosomal damage induced by the *E. oleracea* seed extract, following the protocol described by Fenech (2007) with minor modifications.

Cells were seeded in six‐well plates at a density of 1 × 10^5^ cells/well and incubated for 24 h to allow cell attachment. Cells were then exposed for 3 h to three noncytotoxic concentrations of the extract (62.5, 125, and 250 µg/mL). The following controls were included: negative control (complete culture medium), solvent control (0.1% DMSO), and positive control (100 µM hydrogen peroxide, known genotoxic agent).

After treatment, cells were washed and incubated with cytochalasin B (3.0 µg/mL) for approximately 1.5 cell cycles to block cytokinesis. Cells were subsequently trypsinized, centrifuged (900 rpm, 5 min), and subjected to hypotonic treatment with 0.075 M KCl. Fixation was performed using cold methanol:acetic acid (3:1), repeated twice.

Cell suspensions were dropped onto precleaned glass slides, air‐dried, and stained with Giemsa. Slides were analyzed under a light microscope using a 40× objective.

A total of 3000 binucleated cells were scored per treatment (1000 per replicate) for micronucleus frequency according to the morphological criteria described by Fenech (2007). In addition, 500 cells per replicate (mononucleated, binucleated, and multinucleated) were scored to determine the cytokinesis‐block proliferation index (CBPI), calculated according to the equation proposed by the OECD (2016), providing complementary information on cytotoxicity and potential interference with cell cycle progression.

$$\mathrm{CBPI}=\frac{\left(\mathrm{no}.\;\mathrm{of}\;\mathrm{cells}\;\mathrm{mononucleated}\right)+2\left(\mathrm{no}.\;\mathrm{of}\;\mathrm{cells}\;\mathrm{binucleated}\right)+3\left(\mathrm{no}.\;\mathrm{of}\;\mathrm{cells}\;\mathrm{multinucleated}\right)}{500}$$

#### 2.6.2 Ames Test

The Ames test was performed to evaluate the gene‐level mutagenicity of the *E. oleracea* seed extract. The *Salmonella* Typhimurium tester strains TA98, TA97a, TA100, and TA102, kindly provided by Dr. Bruce Ames (Bruce Ames, University of California, Berkeley), were used in the presence and absence of metabolic activation (S9 mix).

Bacterial cultures were prepared from frozen stocks, inoculated into nutrient broth (Oxoid No. 2), and incubated at 37°C for 16 h under shaking to reach the required density of 1–2 × 10^9^ bacteria/mL. The assay followed the preincubation method described by Maron and Ames (1983), using five extract concentrations selected based on solubility and preliminary toxicity assessed in strain TA100, where toxicity was indicated by a reduction in His^+^ revertant colonies.

For each test condition, the extract (62.5–500 µg/mL) was mixed with 0.5 mL of 0.2 M phosphate buffer (without activation) or 0.5 mL of 4% S9 mix (with metabolic activation), plus 0.1 mL of the bacterial culture. The mixture was incubated at 37°C for 20 min.

The S9 fraction allows the evaluation of whether the extract induces mutagenic effects directly or only after metabolic activation by liver‐derived enzymes. The S9 mix, kept on ice throughout the assay and freshly prepared at the time of use, contained 4% S9 fraction (Moltox—Molecular Toxicology Inc., Boone, NC, USA), 1% 0.4 M MgCl_2_, 1% 1.65 M KCl, 0.5% 1 M glucose‐6‐phosphate, and 4% 0.1 M β‐NADP, along with 50% 0.2 M phosphate buffer (pH 7.4) and 39.5% sterile distilled water (Maron and Ames 1983).

After incubation, 2 mL of top agar supplemented with histidine and biotin was added, mixed, and poured onto glucose minimal agar plates. Plates were incubated at 37°C for 48 h, after which His^+^ revertant colonies were manually counted. All treatments were performed in triplicate.

Strain‐specific positive controls were included to confirm assay performance: 4‐nitro‐*o*‐phenylenediamine (10 µg/plate) for TA98 and TA97a; sodium azide (1.25 µg/plate) for TA100; mitomycin C (0.5 µg/plate) for TA102 (without S9); 2‐aminoanthracene (1.25 µg/plate) for TA98, TA97a, and TA100; and 2‐aminofluorene (10 µg/plate) for TA102 (with S9). Negative (solvent; 100 µL DMSO/plate) and spontaneous controls (no treatment) were included in the experiments.

The mutagenicity index (MI) was calculated for each concentration as the ratio between the number of revertant colonies induced by the extract and that of the negative control (solvent). A sample is considered mutagenic when it produces a statistically significant increase in revertants and an MI of ≥2 at any tested concentration (Mortelmans and Zeiger 2000).

### 2.7 Statistical Analysis

Statistical analyses were performed using GraphPad Prism 7.0 (GraphPad Software). One‐way ANOVA was applied to determine statistical significance, followed by Tukey's multiple comparison test, adopting a significance level of *p* < 0.05. The analysis focused on identifying significant differences between treated groups and the negative control (untreated).

For the Ames test, data were evaluated using the SALANAL statistical software developed by the US Environmental Protection Agency (Monitoring Systems Laboratory, Las Vegas, NV, USA; version 1.0, Research Triangle Institute, RTP, Durham, NC, USA). Analysis followed the statistical model described by Bernstein et al. (1982), consisting of ANOVA followed by linear regression.

## 3 Results

### 3.1 Chemical Characterization of *E. oleracea* Seed Extract

**FIGURE 1:**
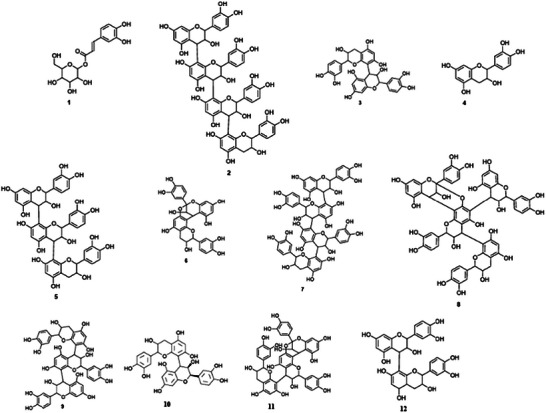
Chemical structures of the compounds listed in Table 1, identified in the açaí seed extract.

LC–ESI‐IT/MS analysis of the 70% ethanolic extract of açaí seeds enabled the chemical annotation of 12 compounds, predominantly belonging to the phenolic acid and proanthocyanidin classes (Table 1). Among the phenolic acids, a glycosylated caffeic acid derivative (*m/z* 341) was annotated, exhibiting a characteristic fragment ion corresponding to the caffeic acid moiety (*m/z* 179). The açaí seed extract was found to be particularly rich in proanthocyanidins with different degrees of polymerization, including B‐type dimers (*m*/*z* 577), trimers (*m/z* 865), and tetramers (*m/z* 1153), as well as A‐type tetramers (*m/z* 1151). Catechin (*m/z* 289), procyanidin C1 (*m/z* 865), and cinnamtannin D1 (*m/z* 863) were also identified based on their characteristic MS*^n^* fragmentation patterns. Overall, the results revealed that the açaí seed extract contains a complex mixture of oligomeric flavan‐3‐ols and phenolic acid derivatives, reinforcing its potential as a rich source of bioactive polyphenols. The chemical structures of the annotated compounds listed in Table 1 are shown in Figure 1.

**TABLE 1:** Compounds annotated in the açaí seed extract (*Euterpe oleracea*), obtained by LC–ESI‐IT/MS.

ID	[M─H]^a^	MS*^n^* ^b^	Compound
1	341	179	Caffeic acid‐hexoside‐dimer/caffeic acid hexoside
2	1153	577; 289	Procyanidin tetramer B‐type
3	577	289	Proanthocyanidin B2
4	289	205	Catechin
5	865	695; 577; 407; 287	Procyanidin trimer
7	1155	983; 865; 577	Procyanidin tetramer
8	1151	863; 577; 289	Procyanidin tetramer A‐type
9	865	739; 500; 285	Procyanidin C1
10	577	407; 287	Procyanidin B2
11	863	863; 575; 289	Cinnamtannin D1
12	577	425; 289	Procyanidin dimer B‐type

^a^Deprotonation.

^b^Multiple‐stage fragmentations.

### 3.2 Determination of TPC in Açaí Seed Extract

The TPC of the *E. oleracea* seed extract was 123.12 ± 5.70 mg GAE/g DW, indicating a substantial concentration of phenolic compounds. The elevated TPC observed in the present study is consistent with the chemical annotation data obtained by LC–ESI‐IT/MS, which revealed the presence of several phenolic acid derivatives and proanthocyanidins, compounds widely recognized for their biological relevance and antioxidant properties (Melo et al. 2021).

### 3.3 Antioxidant Activity

The antioxidant activity of the açaí seed extract was evaluated using the DPPH• radical scavenging assay, and the results are summarized in Table 2. The extract exhibited an EC_50_ value of 11.79 ± 0.9 µg/mL, indicating a strong capacity to neutralize DPPH• radicals. Under the same experimental conditions, the reference antioxidant Trolox presented an EC_50_ of 83.5 ± 3.5 µg/mL. Based on the interpolation of inhibition values in the Trolox calibration curve (*y* = 0.147*x* − 9.8297; *R* ^2^ = 0.9943), the antioxidant activity of the extract corresponded to 3387.35 ± 55 µmol TE/g, confirming its antioxidant potential.

**TABLE 2:** Antioxidant activity of açaí seed extract (*Euterpe oleracea*) determined by the DPPH assay.

	TEAC	EC_50_ (µg/mL)
*E. oleracea* seed extract	3387.35 ± 55	11.79 ± 0.9
Trolox	—	83.5 ± 3.5

*Note*: Results are expressed as mean ± standard deviation.

Abbreviations: DPPH, 2,2‐diphenyl‐1‐picrylhydrazyl; EC50, concentration required to reduce 50% of the DPPH• radical; TEAC, Trolox equivalent antioxidant capacity (µmol Trolox equivalents per gram of extract).

### 3.4 Prebiotic Activity

Given the recognized relationship between polyphenol‐rich plant extracts and modulation of intestinal microbiota, the potential prebiotic effect of the extract was investigated using *L. plantarum*. Bacterial growth and metabolic activity were monitored over 24 h by measuring optical density (OD_600_) and pH variations in different culture media.

**FIGURE 2:**
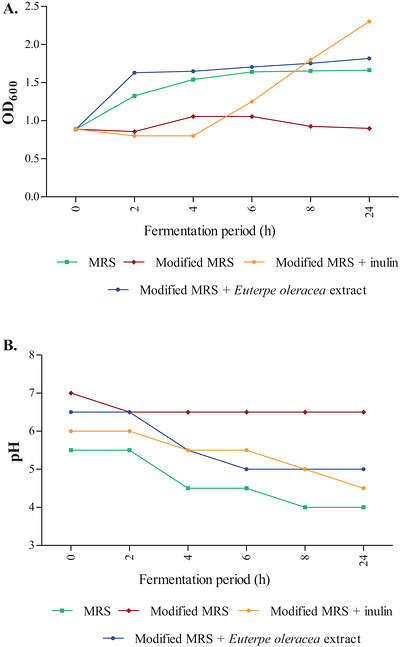
Growth of *Lacticaseibacillus plantarum* in different culture media during in vitro submerged fermentation, represented by (A) optical density (OD_600_) and (B) pH variation at 0, 2, 4, 6, 8, and 24 h. MRS = Man Rogosa and Sharpe broth supplemented with glucose; Modified MRS = glucose‐free MRS broth; Modified MRS + *Euterpe oleracea* extract = modified MRS supplemented with *E. oleracea* (açaí) seed extract (20 g/L); Modified MRS + inulin = modified MRS supplemented with inulin (2 g/L)

As shown in Figure 2A, bacterial growth was significantly influenced (*p* < 0.05) by the composition of the culture medium. In glucose‐free MRS (modified MRS), a lag phase was observed during the first 2 h, followed by only limited growth. In contrast, cultures supplemented with either glucose, inulin, or açaí seed extract rapidly entered the exponential growth phase, showing a progressive increase in OD_600_ over time. In the extract‐supplemented medium, OD_600_ increased from 0.889 at 0 h to 1.818 at 24 h, slightly exceeding the values observed in glucose‐containing MRS (0.889–1.663), suggesting that the extract effectively supported bacterial growth.

Conversely, in the absence of an external carbon source, bacterial growth remained limited. In modified MRS, OD_600_ increased to 1.056 within the first 6 h and subsequently decreased to 0.898 after 24 h (Figure 2A). These results reinforce the importance of accessible fermentable substrates for sustaining bacterial growth.

Changes in pH further supported these observations (Figure 2B). A gradual decrease in pH was observed in both the glucose‐supplemented medium (from 5.5 to 4.0) and the extract‐supplemented medium (from 6.5 to 5.0), consistent with the production of organic acids typical of lactic acid bacterial metabolism. In contrast, the glucose‐free medium exhibited only minor pH variation (from 7.0 to 6.5), indicating reduced fermentative activity. Collectively, these results suggest that the açaí seed extract supports the growth and metabolic activity of *L. plantarum*, indicating its potential as a fermentable substrate for beneficial bacteria.

### 3.5 Cytotoxic Activity

**FIGURE 3:**
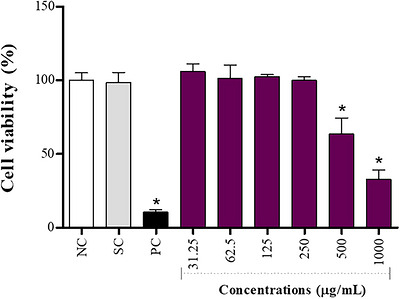
Cell viability of Caco‐2 cells treated for 24 h with different concentrations of açaí seed extract (*Euterpe oleracea*). The asterisk indicates a value significantly different from the negative control (*p* < 0.05, ANOVA followed by Tukey's test). Values are presented as mean ± standard deviation of cell viability (%). IC_50_ (half‐maximal inhibitory concentration) = concentration required to reduce cell viability by 50%, calculated using GraphPad Prism 7.0; NC (negative control) = cells cultured in DMEM supplemented with 20% fetal bovine serum (cell viability: 100 ± 5.2%); SC (solvent control) = cells exposed to 0.1% (v/v) dimethyl sulfoxide (DMSO, cell viability: 98.7 ± 6.8%); PC (positive control) = cells exposed to 50% (v/v) DMSO (cell viability: 10.03 ± 0.8%).

Considering the importance of safety assessment for potential food or nutraceutical applications, the cytotoxicity of the extract was evaluated in Caco‐2 cells. As shown in Figure 3, after 24 h of exposure, a statistically significant reduction in cell viability was observed only at the highest tested concentrations (500 and 1000 µg/mL) when compared with both the negative control and the solvent control (0.1% DMSO). The calculated IC_50_ value was 704.4 ± 41.8 µg/mL, indicating a low cytotoxic potential under the experimental conditions.

### 3.6 Mutagenic Activity

To further investigate the safety profile of the extract, its potential mutagenic effects were assessed using the in vitro micronucleus assay in Caco‐2 cells and the Ames test in *S*. Typhimurium strains TA97a, TA98, TA100, and TA102, performed in the presence (+S9) and absence (−S9) of metabolic activation.

The micronucleus assay results (Table 3) showed that none of the tested concentrations induced a statistically significant increase in micronucleus frequency compared with the negative control. Likewise, no significant differences were observed in the CBPI, indicating that the extract did not interfere with cell proliferation. The CBPI value for the negative control was 1.82 ± 0.02, whereas values for the extract‐treated groups ranged from 1.80 ± 0.02 to 1.84 ± 0.08. In contrast, the positive control (hydrogen peroxide) caused a marked reduction in CBPI (1.65 ± 0.08) and a pronounced increase in micronucleus frequency (32.5 ± 9.5), confirming the sensitivity and validity of the assay.

**TABLE 3:** Micronucleus frequency and cytokinesis‐block proliferation index (CBPI) in Caco‐2 cells after 3 h of treatment with different concentrations of açaí seed extract (*Euterpe oleracea*).

Treatments	MNs	CBPI
NC	4.0 ± 0.7	1.82 ± 0.02
SC	5.0 ± 0.7	1.80 ± 0.05
PC	32.5 ± 9.5^*^	1.65 ± 0.08^*^

µg/mL	*Euterpe oleracea* seed extract	
62.5	5.0 ± 1.0	1.82 ± 0.04
125	4.3 ± 0.5	1.84 ± 0.08
250	4.0 ± 1.2	1.80 ± 0.02

*Note*: Data are expressed as mean ± standard deviation of micronuclei (MNs) per 1000 binucleated cells and CBPI values per 500 cells, per replicate and treatment group. Experiments were performed in triplicate. NC (negative control) = cells cultured in DMEM supplemented with 20% fetal bovine serum; SC (solvent control) = cells exposed to 0.1% (v/v) dimethyl sulfoxide (DMSO); PC (positive control) = cells exposed to 100 µM hydrogen peroxide.

*Statistically different from negative control (*p* < 0.05, ANOVA followed by Tukey's test).

Consistent with these findings, the Ames test results demonstrated that the extract did not induce a statistically significant increase in revertant colonies in any of the tested strains or experimental conditions (Table 4). Furthermore, MI values remained below 2 for all concentrations, indicating the absence of mutagenic activity under the conditions evaluated.

**TABLE 4:** Mean (*M*) and standard deviation (SD) of revertants per plate and mutagenicity index (MI) in *Salmonella* Typhimurium strains TA98, TA100, TA102, and TA97a treated with different concentrations of açaí seed extract (*Euterpe oleracea*), in the absence (–S9) and presence (+S9) of metabolic activation.

	Number of revertants (*M* ± SD)/plate and MI							
	TA98		TA100		TA102		TA97a	
	−S9	+S9	−S9	+S9	−S9	+S9	−S9	+S9
NC	24 ± 3	21 ± 4	108 ± 13	117 ± 18	306 ± 23	209 ± 37	115 ± 21	138 ± 28
SC	26 ± 8	28 ± 2	113 ± 11	112 ± 27	308 ± 36	204 ± 22	127 ± 14	135 ± 11
PC	733 ± 35^*a^	1344 ± 143^*d^	1024 ± 43^*b^	1470 ± 137^*d^	1974 ± 127^*,c^	1253 ± 119^*e^	1203 ± 98^*a^	1377 ± 60^*d^

µg/plate	*Euterpe oleracea* seed extract							
62.5	25 ± 4 (1.02)	24 ± 1 (1.12)	120 ± 11 (1.11)	130 ± 29 (1.11)	319 ± 34 (1.04)	220 ± 21 (1.05)	125 ± 18 (1.08)	145 ± 14 (1.05)
125	23 ± 4 (0.94)	23 ± 4 (1.10)	124 ± 19 (1.14)	139 ± 14 (1.18)	312 ± 21 (1.02)	229 ± 13 (1.10)	128 ± 19 (1.11)	148 ± 22 (1.07)
250	25 ± 1 (1.04)	21 ± 1 (1.00)	110 ± 14 (1.01)	125 ± 13 (1.07)	320 ± 37 (1.05)	221 ± 39 (1.06)	120 ± 26 (1.04)	164 ± 16 (1.18)
375	28 ± 8 (1.17)	23 ± 6 (1.07)	121 ± 12 (1.12)	119 ± 24 (1.02)	299 ± 40 (0.98)	226 ± 27 (1.08)	131 ± 14 (1.14)	136 ± 20 (0.99)
500	26 ± 3 (1.08)	24 ± 9 (1.12)	112 ± 26 (1.04)	103 ± 17 (0.88)	318 ± 28 (1.04)	190 ± 14 (0.91)	114 ± 11 (0.99)	139 ± 37 (1.01)

*Note*: NC (negative control) = dimethyl sulfoxide (DMSO, 100 µL/plate); SC (spontaneous control) = spontaneous reversion rate to each *S*. Typhimurium strain, obtained from plates without treatment; PC (positive controls) = ^a^4‐nitro‐*o*‐phenylenediamine (TA98 and TA97a, 10.0 µg/plate); ^b^sodium azide (TA100, 1.25 µg/plate); ^c^mitomycin C (TA102, 0.5 µg/plate), without S9; ^d^2‐aminoanthracene (TA98, TA100, and TA97a, 1.25 µg/plate); and ^e^2‐aminofluorene (TA102, 10.0 µg/plate), with S9.

Abbreviation: MI, mutagenicity index.

*p < 0.05 (ANOVA).

## 4 Discussion

In the present study, the biological properties and safety profile of açaí seed extract were investigated, with particular emphasis on its antioxidant and prebiotic activities, as well as its cytotoxic and mutagenic potential. *Euterpe oleracea* is widely recognized for its high nutritional value and for containing a diverse range of bioactive compounds associated with beneficial health effects (Laurindo et al. 2023).

According to Laurindo et al. (2023), the chemical composition of açaí pulp comprises approximately 25.5% polyphenols, while the seeds contain an even higher concentration, around 28.3%. Among the main phenolic constituents are cyanidin‐3‐glucoside and cyanidin‐3‐rutinoside, in addition to fatty acids such as lauric, myristic, palmitic, palmitoleic, oleic, and linoleic acids.

In the present study, the *E. oleracea* seed extract exhibited a TPC of 123.12 ± 5.70 mg GAE/g DW, confirming the abundance of phenolic constituents in this agro‐industrial byproduct. This result is consistent with previous reports demonstrating that açaí‐derived matrices, particularly the seeds, represent an important source of polyphenolic compounds (Gordon et al. 2012; G. R. Martins et al. 2022; Laurindo et al. 2023). In agreement with these observations, LC–MS analysis revealed a predominance of proanthocyanidins as the major constituents of the extract. The hydroethanolic extraction system employed in the present study likely favored the recovery of these medium‐polarity polyphenols, contributing to the expressive antioxidant activity observed.

Proanthocyanidins, classified as oligomeric flavan‐3‐ols, are structurally characterized by a high density of hydroxyl groups capable of donating hydrogen atoms or electrons to neutralize reactive species. In addition, these compounds can stabilize phenoxyl radicals through resonance mechanisms and exert metal chelating activity, which further enhances their antioxidant potential (Yang et al. 2018; Gulcin 2025). Synergistic interactions between proanthocyanidins and other phenolic constituents identified in açaí matrices, including anthocyanins and low‐molecular‐weight flavonoids, may also contribute to the overall redox‐modulating capacity of the extract, reinforcing the relevance of its phenolic composition to the biological activities observed.

Consistent with this chemical profile, the antioxidant potential of the 70% ethanolic extract was confirmed by the DPPH radical scavenging assay, which demonstrated a high antioxidant capacity (3387.35 ± 55 µmol Trolox equivalents/g extract) and a low EC_50_ value (11.79 ± 0.9 µg/mL). Notably, the extract exhibited greater radical scavenging efficiency than the reference antioxidant Trolox, which presented an EC_50_ of 83.5 ± 3.5 µg/mL, indicating a pronounced ability to neutralize DPPH• radicals.

The results obtained here are consistent with previous reports describing the antioxidant activity of *E. oleracea* seed extracts obtained using different solvents and analytical approaches (Rodrigues et al. 2006; Barros et al. 2015; G. R. Martins et al. 2020, 2022; Previtalli‐Silva et al. 2024). Melo et al. (2021), for example, reported significant in vitro antioxidant activity of açaí seed extracts against DPPH (622.81 µmol/g) and ABTS radicals (763.09 µmol TEAC/g). Similarly, G. R. Martins et al. (2020) observed that a methanolic extract of açaí seeds exhibited an EC_50_ of 16.95 µg/mL in the DPPH assay, with a TEAC value of 3835.44 µmol Trolox/g extract and an ORAC value of 4082.16 µmol Trolox/g. Previtalli‐Silva et al. (2024) reported even stronger antioxidant activity for hydroalcoholic extracts, with an EC_50_ of 6.03 ± 0.28 µg/mL in the DPPH assay, a FRAP value of 2553.67 ± 0.01 µmol TE/g in the FRAP assay, and an IC_50_ of 69.84 ± 0.32 µg/mL in the ABTS assay.

Variations among studies may arise from differences in extraction solvents, phytochemical composition, geographical origin, fruit maturation stage, and experimental conditions. Nevertheless, the available evidence consistently supports that *E. oleracea* seeds represent a valuable source of antioxidant compounds, reinforcing their potential for functional and biotechnological applications.

Beyond its pronounced antioxidant activity, the 70% ethanolic extract of *E. oleracea* seeds also promoted the growth of *L. plantarum*, a well‐established probiotic widely applied in food and pharmaceutical industries (Behera et al. 2018). This effect may be associated not only with the presence of fermentable substrates but also with the phenolic profile identified by LC–MS, particularly the predominance of proanthocyanidins.

Previous studies have demonstrated that flavan‐3‐ols and procyanidins may positively influence gut microbiota composition and stimulate the production of short‐chain fatty acids (SCFAs), reinforcing the relevance of polyphenol‐rich substrates in intestinal homeostasis and host–microbiota interactions (Ou et al. 2014; Márquez Campos et al. 2019). In addition, anthocyanins and related phenolic compounds have been associated with enhanced microbial enzymatic activity, modulation of microbial metabolic pathways, and selective inhibition of pathogenic microorganisms (Wang et al. 2022).

From a gastrointestinal perspective, highly polymerized phenolic compounds generally exhibit limited absorption in the upper gastrointestinal tract and may therefore reach the colon in greater amounts, becoming more available for microbial biotransformation. Under these conditions, microbial conversion of flavan‐3‐ols and procyanidins may generate lower molecular weight metabolites capable of influencing bacterial metabolic activity and fermentation dynamics (Niwano et al. 2022).

To the best of our knowledge, studies specifically evaluating the effects of hydroethanolic extracts obtained from *E. oleracea* seeds on probiotic bacterial growth remain limited, highlighting the relevance of the present findings and the potential biotechnological value of this agro‐industrial byproduct.

Alqurashi et al. (2017), using a batch fermentation model with human fecal microbiota, demonstrated that digested açaí pulp modulated microbial composition by increasing the production of SCFAs such as acetate, propionate, and butyrate. Additionally, fermentation supernatants retained antioxidant activity and inhibited hydrogen peroxide‐induced DNA damage in HT‐29 colon cells, as evidenced by the comet assay.

H. V. Freitas et al. (2021) showed that beverages formulated with açaí juice maintained the viability of *Lacticaseibacillus casei* for up to 42 days under refrigeration, indicating the potential of açaí as a symbiotic matrix. Similarly, Reges et al. (2024) reported that a symbiotic açaí juice enriched with gluco‐oligosaccharides, dextran, and *Bifidobacterium breve* increased microbial diversity and enhanced the production of fermentation metabolites after incubation with human fecal microbiota. More recently, Loubet Filho et al. (2025), using an in vivo model, demonstrated that supplementation with lyophilized açaí pulp promoted shifts in intestinal microbiota composition and SCFA production, including an increased relative abundance of beneficial genera such as *Lactobacillus* and *Bifidobacterium*.

Given that prebiotic compounds exert their biological effects through interactions with the intestinal epithelium, the cytotoxic and mutagenic potential (micronucleus assay) of the extract was evaluated in Caco‐2 cells. This evaluation was complemented by the Ames test to determine its ability to induce gene mutations and further support the safety assessment of the extract.

Caco‐2 cells, derived from human colorectal adenocarcinoma, differentiate into monolayers exhibiting enterocyte‐like characteristics, including tight junctions, microvilli, enzymes, and transporters. Consequently, they are widely used as an in vitro model for investigating intestinal absorption and the biological effects of bioactive compounds on the intestinal barrier (Srinivasan et al. 2015).

The results demonstrated that the 70% ethanolic extract of açaí seeds exhibited low cytotoxicity in Caco‐2 cells and showed no mutagenic effects in either the micronucleus or Ames assays. The combined use of these complementary approaches provides a broader assessment of the genetic safety of the extract.

Silva et al. (2014) reported that hydroalcoholic extracts obtained from seeds, peels, and fruits of *E. oleracea* did not exhibit cytotoxic effects in Caco‐2, HT‐29, or MDA‐MB‐468 cells. Interestingly, the extracts significantly reduced cell viability in the MCF‐7 breast cancer cell line, suggesting possible selective cytotoxicity against specific tumor types. G. R. Martins et al. (2020) demonstrated that açaí seed extract did not induce cytotoxic effects in epithelial cells (LLC‐MK2) or macrophages and exhibited cytoprotective activity against oxidative stress, which was associated with its high antioxidant capacity. In addition, antimicrobial activity was observed against Gram‐positive bacteria and *Candida albicans*.

Similar results have been reported for derived and processed açaí products. da Silva et al. (2025) evaluated a beverage produced from roasted açaí seeds and observed low cytotoxicity, with cell viability above 90%, along with cytoprotective effects in Caco‐2 cells after gastrointestinal simulation. Likewise, Cordeiro et al. (2026) demonstrated that digested extracts from pulps of different *Euterpe* species generally exhibited low cytotoxicity in Caco‐2 cells, with *E. oleracea* maintaining the highest cell viability even at elevated concentrations.

Regarding mutagenicity, Zimmer et al. (2025) evaluated the genetic safety of a standardized *E. oleracea* seed extract (TI‐35) and reported no mutagenic activity in the Ames test. Furthermore, the extract did not induce skin irritation or genotoxic effects in human cellular models.

With respect to other açaí‐derived products, Marques et al. (2016) demonstrated that açaí oil did not induce genotoxicity in leukocytes, liver, bone marrow, or testicular cells in rodents, based on comet and micronucleus assays. In a subsequent investigation, Marques et al. (2017) reported the absence of cytotoxic and genotoxic effects of açaí oil in human lymphocytes and HepG2 hepatic cells, although no chemoprotective effects were observed against genotoxic agents such as methanesulfonate and benzo[*a*]pyrene.

Taken together, the findings of the present study expand the current knowledge regarding *E. oleracea* seed extracts, particularly by integrating functional and safety evaluations of a proanthocyanidin‐rich hydroethanolic extract, thereby supporting the potential valorization of this agro‐industrial byproduct as a source of bioactive compounds for functional and nutraceutical applications.

## 5 Conclusion

In conclusion, the results of this study demonstrate that the 70% ethanolic extract of *E. oleracea* seeds presents a relevant combination of biological activity and safety, supporting its potential as a functional bioactive ingredient. The extract exhibited pronounced antioxidant capacity associated with a phenolic‐rich chemical profile dominated by proanthocyanidins, compounds widely recognized for their health‐promoting properties. In addition, the extract was able to stimulate the growth of *L.; plantarum*, indicating its potential prebiotic activity. Importantly, safety assessments revealed low cytotoxicity in Caco‐2 intestinal cells and no evidence of mutagenicity in either the micronucleus assay or the Ames test, reinforcing the favorable toxicological profile of the extract. Taken together, these findings highlight *E. oleracea* seeds as an abundant and underutilized agro‐industrial residue and as a promising source of bioactive compounds with potential applications in functional foods, nutraceuticals, and microbiota‐targeted biotechnological products. Furthermore, the valorization of this byproduct contributes to sustainable resource management and circular bioeconomy strategies in the açaí production chain.

Future studies involving in vivo models and more complex gut microbiota systems are warranted to further elucidate the mechanisms underlying the observed biological effects and to support the development of innovative health‐promoting products derived from açaí seed.

## Author Contributions

**Marcelo Pereira da Silva Junior**: investigation, data curation, formal analysis, writing – original draft. **Amanda de Jesus Alves Miranda**: investigation, resources. **Ana Laura Gagliardi**: investigation, formal analysis. **Isabelle Guerreiro Moreira**: investigation, formal analysis. **Maria Eduarda Santos Camaceti**: investigation, formal analysis. **Mateus Kawata Salgaço**: investigation, formal analysis. **Kátia Sivieri**: supervision, formal analysis, funding acquisition. **Cláudia Quintino da Rocha**: investigation, resources. **Flávia Aparecida Resende**: conceptualization, methodology, supervision, writing – review and editing, funding acquisition. All authors contributed to the revision of the manuscript and approved the final version.

## Conflicts of Interest

The authors declare no conflicts of interest.

## Data Availability

Data will be made available on request.
